# Fitness-for-Service Assessment of Dent Defects on Steel Energy Pipelines: Evaluation Criteria, Integrity Prediction, and Future Challenges

**DOI:** 10.3390/ma19122616

**Published:** 2026-06-17

**Authors:** Yunfei Huang, Jianrong Tang, Dong Lin, Mingnan Sun, Jie Shu, Wei Liu, Xiangqin Hou

**Affiliations:** 1 Institute of Safety, Environmental Protection, and Technical Supervision, PetroChina Southwest Oil & Gasfield Company, Chengdu 610095, China; 2 PetroChina Southwest Oil & Gasfield Company, Chengdu 610094, China; 3 School of Mechatronic Engineering, Southwest Petroleum University, Chengdu 610500, China; 4 School of Civil Engineering and Geomatics, Southwest Petroleum University, Chengdu 610500, China

**Keywords:** steel energy pipelines, dent defects, evaluation criteria, integrity prediction, bibliometric analysis

## Abstract

Due to climate change, corrosive conditions, and hydrogen-rich environments, steel energy pipelines inevitably develop a variety of defects. These deficiencies compromise pipeline safety and reliability, and neglecting them may result in pipeline leaks, fractures, and even potentially catastrophic explosions. Although a considerable body of literature reviews the effects of metal-loss defects like corrosion and cracks on pipeline safety and reliability, the impact of geometric deformation, like dents, lacks a comprehensive review. This work employs a hybrid systematic literature review (SLR) and bibliometric analysis (BA) to investigate the current research status of pipeline dent assessment. Four questions are answered: (1) What are the publication distribution characteristics, active journals, production organizations, and production authors related to research on pipeline dents? (2) What criteria have been employed for evaluating the pipeline dent? (3) From what perspective has the integrity of dented pipelines been assessed, and what research approaches have been used? (4) What are the future challenges and prospects of pipeline dent studies? The findings demonstrate that depth-, strain-, and damage-based evaluation criteria are widely employed to assess pipeline dents, each with merits and limitations. Despite the simplicity and ease of use of depth- and strain-based criteria, they are prone to underestimation flaws. In contrast, damage-based criteria, which consider multiple factors, are limited by their complexity and high computational resource requirements. The reliability of dented pipelines is predicted with remaining strength, fatigue life, and failure pressure using theoretical modeling, experimental testing, numerical simulation, or a combination of these methods. Future dent studies should involve refining numerical models, full-scale testing under varied loading conditions, and integrating advanced sensing techniques for real-time inspection.

## 1. Introduction

Due to their favorable safety and economic profiles, pipelines are a well-established transportation medium for oil and gas supplies [1,2]. With the need for energy transition and a net-zero emissions proposal, pipelines have also been used to transport clean energy, such as hydrogen, to meet the growing demand for clean energy [3]. However, oil, natural gas, and hydrogen are flammable and explosive materials, and pipelines usually have the characteristics of a large transmission volume and high pressure. Once pipeline leakage occurs, severe accidents such as fire and explosion may be induced, with catastrophic consequences [4]. Therefore, ensuring the integrity of pipelines is of considerable significance to their safe operation. However, pipeline failure accidents stem from a multitude of factors [5,6], including corrosion (both internal and external), geohazards, construction issues and material flaws, as well as third-party activities. These factors may result in various pipeline defects, negatively affecting structural integrity and reliability [7,8,9]. Hence, a thorough understanding of pipeline imperfections and their assessment methodologies is crucial to devising strategies to enhance steel pipelines’ reliability and durability.

**Figure 1:**
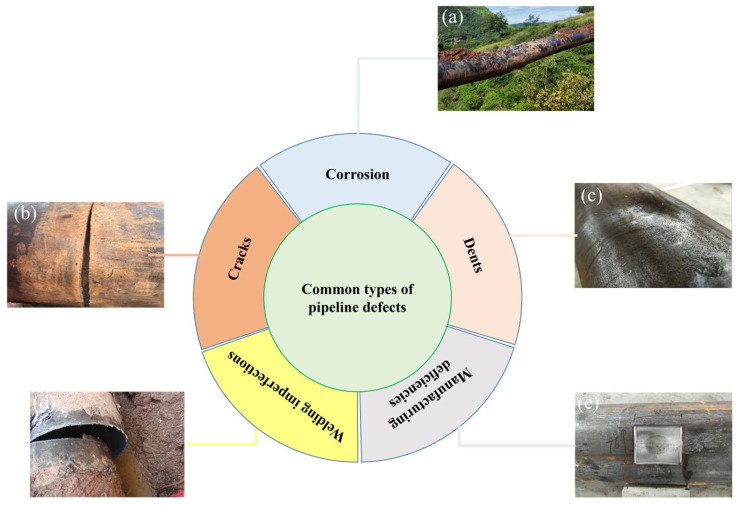
Different types of pipeline defects: (**a**) pipeline corrosion; (**b**) pipeline crack; (**c**) pipeline dent; (**d**) welding imperfection in a pipeline; (**e**) manufacturing deficiency in a pipeline.

The presence of defects in pipelines presents a significant challenge to their safety operations. Swift identification and effective resolution of these issues are imperative to mitigate potential risks and guarantee the lifespan of pipeline infrastructures. Figure 1 illustrates various types of defects occurring on pipelines, including corrosion, cracks, dents, welding imperfections, manufacturing deficiencies, etc. Different from metal loss defects like corrosion and cracks, dents are the most common geometric deformation on pipelines [10]. As shown in Figure 1c, a dent is a permanent inward plastic deformation of the pipe’s cross-section that results in a reduction in the diameter [11]. Pipelines can sustain dents resulting from improper forming procedures during manufacturing, mishandling during transportation, installation, and backfilling operations in the construction period, geological disasters, and third-party activities [12]. The presence of these dents can cause local strain/stress concentration, leading to potential crack initiation, fatigue damage, and pigging failures, ultimately reducing pipeline reliability and in-service performance [13]. Several parameters, such as dent depth, dent length, and curvature radius, can impact the safety of pipelines [14]. Note that unconstrained dents exhibit spring back and re-rounding behaviors (tendency to restore the initial pipeline geometry) under internal pressure, causing changes in the geometric parameters of the dents [15]. 

Regarding metal loss defects like corrosion and cracks, researchers have extensively reviewed their effects on pipeline safety and integrity [16,17,18,19,20,21,22,23,24,25]. In contrast, due to the complex elastic–plastic deformation, rebound behavior, and internal pressure interactions involved, the evaluation of pipeline dents differs significantly from other defects such as corrosion and cracks, which presents more analytical challenges. Therefore, it is necessary to review and organize the scattered findings from various studies on pipeline dents to provide support for the integrity management of dented pipelines. As far as the authors are aware, however, there is a lack of a consolidated summary and systematic review of the efforts in pipeline dents. Thus, this work aims to review pipeline dent-related studies spanning two decades. Guided by the principle that research questions must be clear, specific, and directly aligned with the research objective, we seek to address the following research questions in this work:

(1) What are the publication distribution characteristics, active journals, production organizations, and production authors related to research on pipeline dents?

(2) What criteria have been employed for evaluating the pipeline dent?

(3) From what perspective has the integrity of dented pipelines been assessed, and what research approaches have been used?

(4) What are the challenges and prospects of pipeline dent studies?

The core innovation of this review lies in its combination of SLR and BA, focusing on dent defects in steel energy pipelines, filling the gap in existing reviews that only focus on corrosion, cracking, and other metal loss defects and ignore dent deformation defects; in addition, this work systematically reviews three dent assessment criteria (based on depth, strain, and damage), and compares their advantages, limitations, and application scope in detail, which has not been completed in previous literature reviews.

The review’s structure is as follows: The research framework and methodology are outlined in Section 2. The results of the bibliometric analysis related to RQ1 are presented in Section 3, which also addresses RQ2 and RQ3. Finally, this paper concludes in Section 4 and discusses the prospects and challenges associated with pipeline dent studies in response to RQ4.

## 2. Review Framework and Methodology

**Figure 2:**
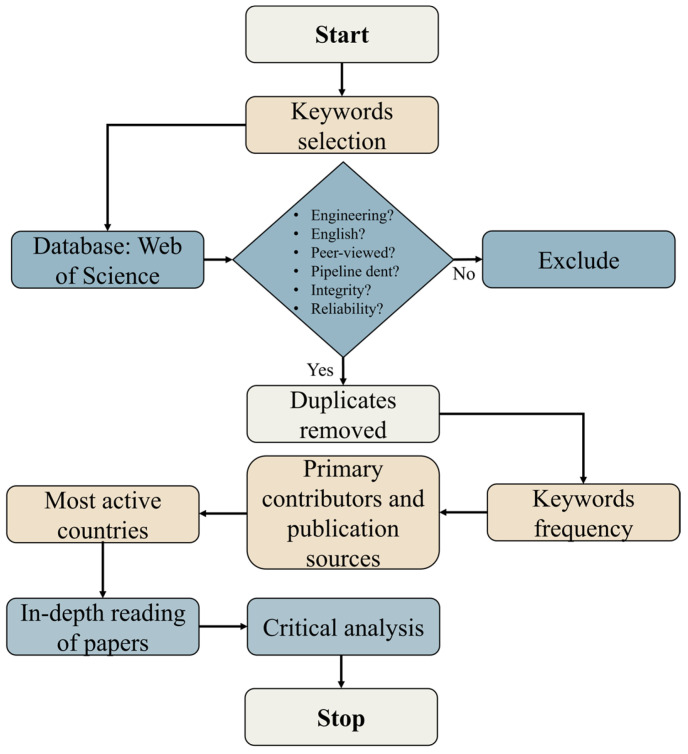
Flowchart of the hybrid BA-SLR methodology.

Compared with traditional narrative review (TNR) techniques, SLR provides a standardized review process that details the selection, scanning, and evaluation processes, thereby reducing bias and enhancing reproducibility [26,27]. Complementing this, BA is a visualization tool that can identify emerging trends, key players, and critical research areas in the literature [28,29]. This work integrated a BA-SLR hybrid method serving as a rigorous analytical vehicle, as presented in Figure 2, to evaluate the pipeline dent research. The framework begins with a rigorous search strategy to retrieve the preliminary literature related to pipeline dents, followed by the systematic screening of retrieved publications against the designed inclusion/exclusion criteria. During screening, all abstracts of the literature are carefully analyzed to evaluate the quality of the materials. After screening and quality assessment, a BA is employed in the selected literature to understand the publication time, journal, geographic location, prominent authors and their relationships, as well as commonly used keywords. An in-depth reading and a critical analysis are conducted with the aim of understanding the evaluation criteria for pipeline dents and the perspectives and methods for assessing the integrity of dented pipelines. The final step is to discuss the prospects and challenges associated with pipeline dent research. The specific process is described in Section 2.1, Section 2.2, Section 2.3 and Section 2.4.

### 2.1. Search Strategy

One of the distinctive features of SLR is its rigorous search process, setting it apart from traditional literature reviews. A tailored search strategy for the Web of Science (WoS) Core Collection database (Editions: All) is employed to identify pertinent literature material for SLR. WoS’s strict journal selection process ensures the quality of literature used for SLR, without including other databases such as Scopus, Engineering Village, Google Scholar, etc., in order to avoid duplicate searches and reduce the interference of irrelevant literature, thus focusing on high-quality journal articles closely related to pipeline dent defect assessment. First, the abstracts of numerous academic papers related to the subject matter are analyzed, as summarized in Table 1, to identify the most used keywords. Subsequently, the search strings utilized in the WoS database are derived, as shown in Table 2. It is noted that the search limits the period between 1 January 2005 and 31 December 2024. Three hundred and eleven (311) publications were determined during the preliminary search stage.

### 2.2. Criteria for Inclusion and Exclusion

After retrieving preliminary literature related to the review topic, it is essential to assess its applicability. The inclusion/exclusion criteria for this study are based on the Preferred Reporting Items for Systematic Reviews and Meta-Analyses (PRISMA) statement [30], which is a widely accepted SLR guideline. The PRISMA statement requires clear inclusion and exclusion criteria for the review, thus the inclusion and exclusion criteria for this SLR are: limited to selected papers in the “engineering field” related to “safety assessment and reliability prediction of dented pipelines”, excluding irrelevant materials; English is the primary language of the global academic community, therefore it is included in all peer-reviewed conference proceedings and journal articles published in English, excluding any other types of publications. Table 3 lists the preliminary criteria for literature inclusion and exclusion according to the PRISMA statement. Based on the proposed inclusion and exclusion criteria, 294 papers were included for further quality assessment.

### 2.3. Quality Assessment

In addition to the inclusion/exclusion criteria, a rigorous evaluation of literature quality is crucial to ensure the quality of selected materials and relevance to the review topic. For this purpose, all literature abstracts are carefully reviewed and analyzed to determine the material’s quality using the following criteria: the research must focus on the evaluation criteria for pipeline dent defects and the structural integrity prediction of dented pipelines (including residual strength, fatigue life, failure pressure, etc.); the literature is limited to peer-reviewed journal articles or conference papers supported by experimental verification, numerical simulation, or theoretical modeling. After screening and quality evaluation, one hundred and twelve (112) papers were ultimately selected from 294 articles.

**Figure 3:**
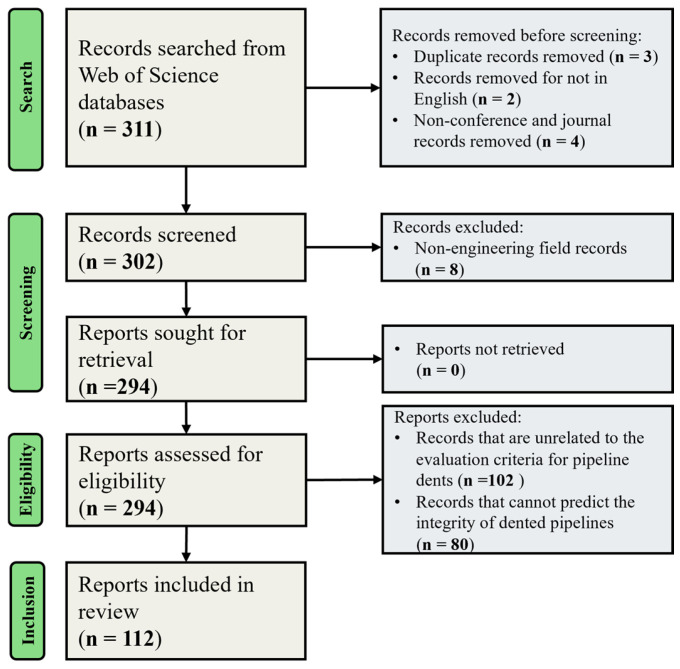
The selection procedure of studies included in the review.

The specific literature selection process (Figure 3) is as follows:

(1) Initial identification: 311 records were retrieved from Web of Science Core Collection (2005–2024).

(2) Duplicate removal: 3 duplicate records were excluded.

(3) Language and publication type screening: 2 non-English records and 4 non-journal/conference records were excluded.

(4) Topic relevance screening: 8 non-engineering field records, 102 records irrelevant to dent evaluation criteria, and 80 records unable to predict dent pipeline integrity were excluded.

(5) Final inclusion: 112 papers for SLR and BA.

### 2.4. Bibliometric Analysis

A BA is performed to assess the distribution of the collected 112 study materials, including several vital dimensions: timeline distribution, frequently employed keywords, geographic regions, collaborative authorship, and targeted publications journals. In this work, BA is implemented by VOSviewer version 1.6.21 (https://www.vosviewer.com/) and Scimago Graphica (https://www.graphica.app/) software, accessed on 11 June 2026.

## 3. Results and Discussions

This section presents the study’s findings with selected 112 publications related to the following questions: (1) What are the publication distribution characteristics, active journals, production organizations, and production authors related to research on steel pipeline dents? (2) What criteria have been employed for evaluating the steel pipeline dent? (3) From what perspective has the integrity of dented steel pipelines been assessed, and what research approaches have been used?

### 3.1. RQ1: What Are the Publication Distribution Characteristics, Active Journals, Production Organizations, and Production Authors Related to Research on Steel Pipeline Dents?

To address RQ1, a BA was conducted to provide insights into the distribution of papers, including publication time, journals, geographical location, primary authors and their connections, and frequently used keywords.

#### 3.1.1. Temporal Distributions and Frequently Used Keywords of Publications

**Figure 4:**
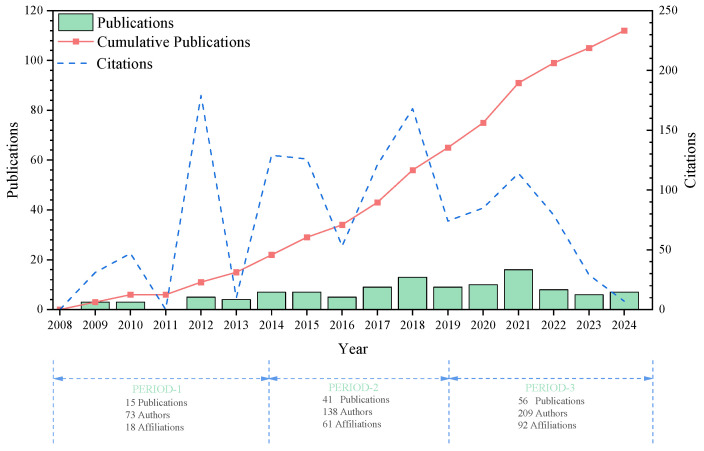
Temporal trend of number for publications.

The temporal evolution of publications can serve as an indicator of the popularity of specific scientific research topics. As illustrated in Figure 4, a steady and robust increase in published articles has been observed over the preceding two decades. During the first period (2008–2013), 15 papers were published by 73 authors from 18 affiliated institutions, with citation counts higher than the number of publications. This may indicate that the few papers published earlier received continued and stable attention. A total of 41 articles were written by 138 researchers from 61 institutions in the second period (2014–2018), indicating a growing interest in the topic. However, there have been significant fluctuations in citation rates, reflecting the replacement of research hotspots: the publication of a considerable achievement may trigger a wave of citation peaks, followed by a gradual decline in popularity, and then breakthrough research may drive another citation peak. Finally, in 2019–2024, 209 researchers from 92 affiliated institutions published 56 manuscripts. The decline in citations after 2021 may be due to the lagging nature of these data statistics. The papers from these recent years have not had enough time to accumulate citations. This temporary reduction does not indicate a decrease in the popularity of pipeline dent research but rather reflects the inherent time lag in citation accumulation. Against the common global backdrop of sustained growth in scientific publications, the fact that the publication growth rate in this research field is higher than the average level of the broader pipeline engineering discipline provides strong empirical support for the conclusion that research attention to this topic is steadily increasing. These findings collectively suggest that the energy transportation industry is demonstrating escalating interest in the safety assessment of pipeline dents, and further indicate that the field of dented pipeline safety assessment remains in a phase of rapid and dynamic development.

**Figure 5:**
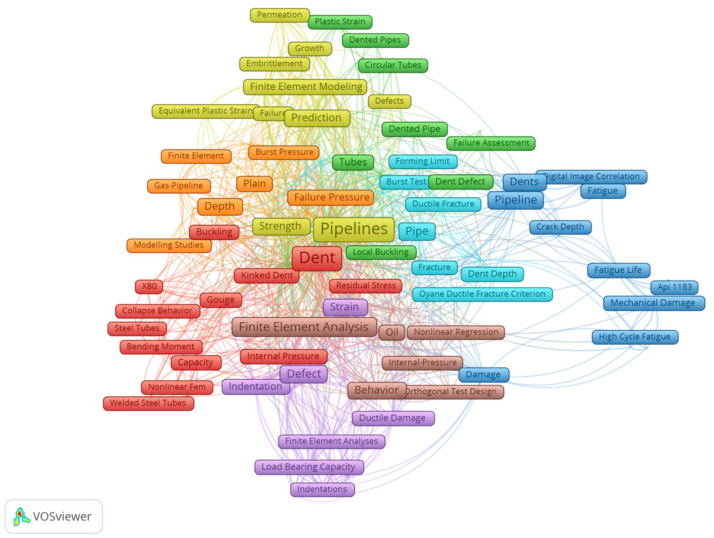
Frequent keywords in the field.

On the other hand, the prevalence of keywords enables us to describe the developmental trajectory of research. Two distinct types of keywords are usually distinguished: author and index. Journal editors select author keywords to describe the paper’s contents, while index keywords are drawn from external databases that are widely available online. BA results reveal that the authors employed a total of 274 keywords, supplemented by 89 keywords, to identify 363 frequently used terms. The distribution of these terms is shown in Figure 5, where each rounded rectangle (node) corresponds to a particular keyword, with its size indicating the frequency of its occurrence. The color of processes represents a collaborative cluster, while the curve signifies the relationship between different keywords. Notably, the most commonly appearing keywords in our analysis include “dent”, “pipelines”, and “pressure”. This observation suggests that the studies primarily focus on the safety and dependability of pipelines containing dent defects under internal pressure.

#### 3.1.2. Cooperative Contributors and Sources of Publications

**Figure 6:**
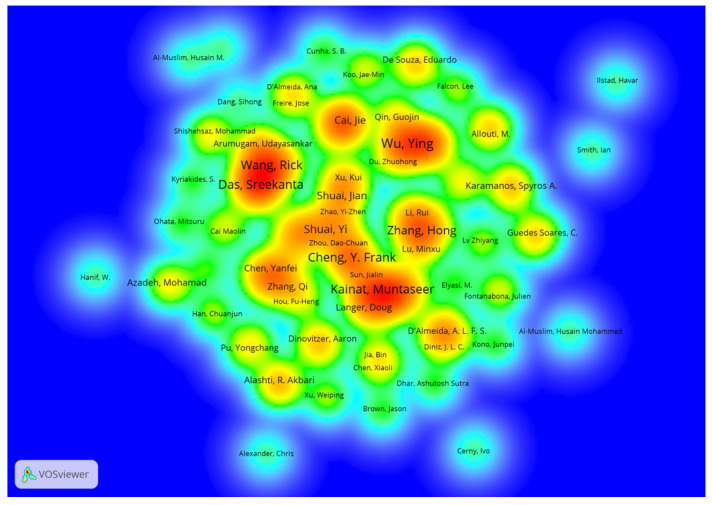
Authors actively involved in the field of pipeline dent assessment.

**Figure 7:**
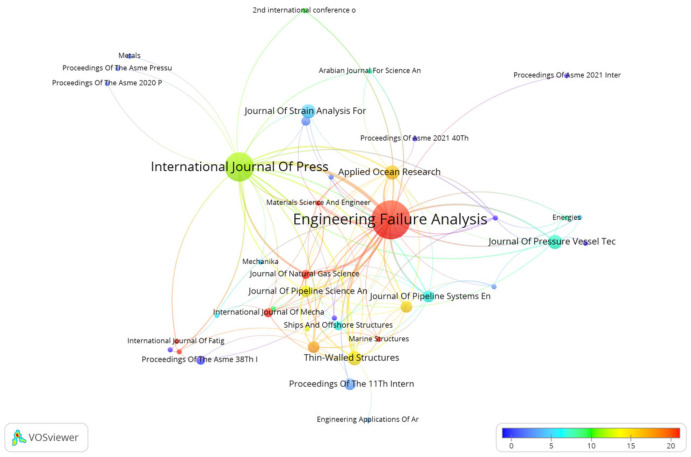
Active journals on safety and reliability of pipelines with dent defects.

According to the BA outcome, 260 authors from 105 research institutions have contributed to publications across 54 academic journals and conference proceedings. The principal authors and publishing organizations are demonstrated in Figure 6 and Figure 7. Figure 6 reveals that the authors’ cooperation network can be categorized into three key clusters, interconnected through Cheng Y. Frank of the University of Calgary in Canada, Das Sreekanta from the University of Windsor in Canada, and Wu Ying at Southwest Petroleum University in China, respectively. The most prolific author is Wu Ying from Southwest Petroleum University, who boasts eight publications, followed by Muntaseer Kaint from Enbridge Pipelines Inc., Canada, Das Sreekanta and Ghaednia Hossein from the University of Windsor, Rick Wang from TransCanada Pipeline Corporation (450 1 Street SW, Calgary, Alberta, T2P 5H1, Canada), Peng Zhang from Southwest Petroleum University and Cheng Y. Frank from University of Calgary, ranking second for their paper amounts (7). Allouti Mustapha, Pluvinage Guy, and Schmitt Christian have received considerable attention due to their high citation rates in publications.

Figure 7 provides clear evidence from the total number of citations and publications, indicating that Engineering Failure Analysis is a preeminent journal in the domain of pipeline dent evaluation, with 17 publications and 422 citations. Following closely behind are the International Journal of Pressure Vessels and Piping, with twelve publications and 128 citations, and Thin-walled Structures, with four publications and 57 citations. Other notable journals in this field include the Journal of Pipeline Science and Engineering, the Journal of Pipeline System Engineering and Practice, and the Journal of Pressure Vessel Technology. The International Pipeline Conference, organized by the American Society of Mechanical Engineers (ASME), also features many publications related to this domain.

#### 3.1.3. Country Distributions of Publications

**Figure 8:**
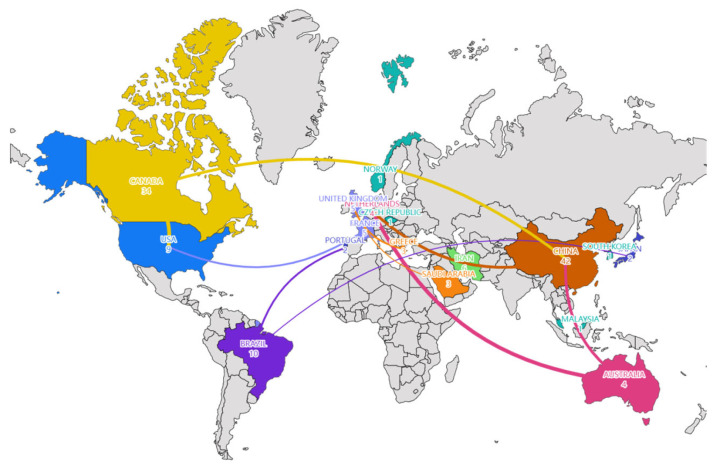
Geographical distribution of publications.

According to the institutional addresses of the publications, a total of 17 countries have been identified, as illustrated in Figure 8. As is clear, the country with the highest number of publications is China (42), followed by Canada (34), Brazil (10), and the United States of America (9). These countries are mainly located in America and Asia, occupying an important position in the production, transportation, and consumption of global oil and gas. Hence, it is natural that these regions prioritize pipeline safety and reliability, resulting in a substantial number of publications related to pipeline defect assessment.

### 3.2. RQ2: What Criteria Have Been Employed for Evaluating the Steel Pipeline Dent?

**Figure 9:**
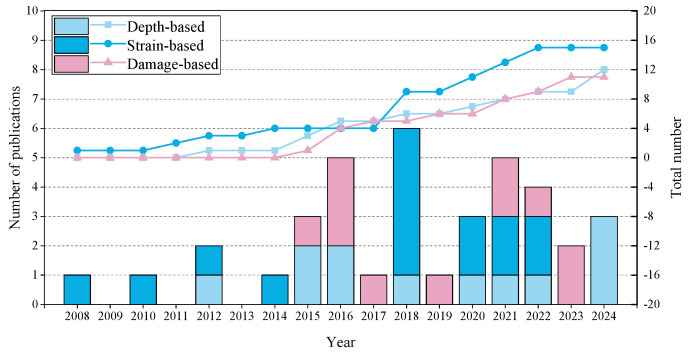
Annual publications.

A thorough examination of 112 publications used in the BA was conducted to answer RQ2. The BA and SLR are based on the same final dataset (112 papers) after systematic screening, rather than the initial 311 retrieved records. The findings show that the industry-accepted dent evaluation criteria typically evaluate the severity of a dent in a pipeline based on any depth, strain, or damage metrics. If the metric exceeds a predefined threshold, the pipeline is damaged and requires repair or replacement. Figure 9 illustrates the trajectory of publications that employed these metric-based criteria to assess pipeline dents, where the line with markers represents the cumulative number of publications, and the bar chart indicates the annual publication count. It is evident that the volume of published works has increased since 2008, and this trend is expected to persist in the future. The following sub-sections detail the application of these metric-based criteria in the publications.

#### 3.2.1. Depth-Based Dent Assessment on Steel Pipelines

**Figure 10:**
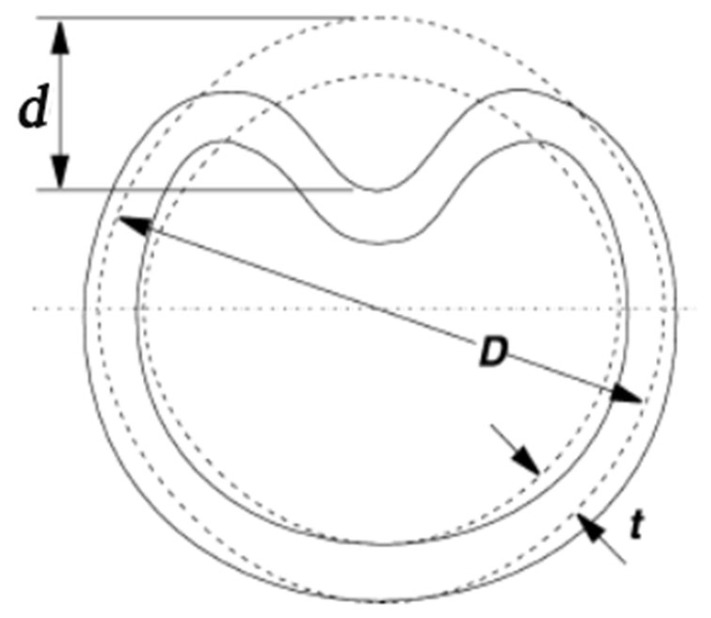
Geometric diagram of a pipeline dent, where *d*, *D* and *t* represent the dent depth, pipe outer diameter, and pipe wall thickness, respectively.

Depth-based criterion is a methodology for determining a dent’s severity by measuring the pipe’s radial deformation distance at the defect, as shown in Figure 10. Allouti et al. [31] employed experimental and numerical approaches to examine the impact of dent depth on pipeline burst pressure. They performed indentation and burst tests to validate a commonly used empirical rule that the critical dent depth is equivalent to 10% of the pipe’s outer diameter (*D*). The outcomes showed that a single dent had an insignificant impact on the pipeline’s burst pressure, indicating that the critical depth (10% *D*) rule is overly conservative. Ghaednia et al. [32] experimentally examined the influence of dent depth and operating pressure on the burst capacity of pipelines containing dent–crack defects. Their findings revealed that a combination of a 12% *D* dent depth and a 4 mm or deeper crack depth could significantly reduce the pipe’s pressure-bearing capacity, with a maximum decrease of up to 38%. In a recent investigation, Yang et al. [33] employed numerical simulations to develop a failure criterion and a critical dent-depth calculation method for corroded dented pipelines. They explored the impact of several key factors on critical dent depth. Finally, the team formulated a semi-empirical prediction equation for critical dent depth based on numerical results. To make an accurate prediction of dent depth, a machine learning (ML) model based on the Radial Basis Function (RBF) neural network was constructed by Jia et al. [34], incorporating dent depth data, and it was found to have higher prediction accuracy. A finite element (FE)-based phase field method was developed by Zhao and Cheng [35] to predict the threshold dent depth for crack initiation in dented pipelines considering hydrogen impact, revealing that increased hydrogen concentration and internal pressure reduced the threshold dent depth for crack initiation. Feng et al. [36] proposed a new method with Bayesian statistical theory and neural networks to calculate dent depth based on the pipe’s bending strain. This method is verified to be accurate, with a mean relative error of 2.44%. Zhang and Lan [37] proposed a critical depth calculation model for steel pipelines with kinked dents. By conducting tests and simulations, they analyzed the factors affecting the pressure-bearing capacity of kinked dented pipelines. Wu et al. [38] derived the accurate displacement profiles of dent defects and compared various methods for their ability to simulate and calculate the actual depth of dents. The results indicate that the modified cubic spline interpolation is the most precise approach for estimating dent depth. Huang et al. [39,40,41] numerically simulated the mechanical behavior of dented pipelines under internal pressure, focusing on the phenomena of spring-back and re-rounding. The results reveal that both spring back and re-rounding behaviors significantly impacted the variation in dent depth and suggest that they are crucial considerations when employing a depth-based dent assessment method. This finding is also supported by Zhao et al. [42].

#### 3.2.2. Strain-Based Dent Assessment on Steel Pipelines

The strain-based assessment criterion for pipeline dents involves determining strain levels within the dented region. A widely adopted approach for estimating dent strain, as depicted in Table 4, was advocated in Appendix R of the ASME B31.8 code [43]. Rafi et al. [44] employed full-scale tests and FE analyses to validate the strain-based dent assessment criterion in the ASME B31.8 code and its underlying assumptions. The results indicate that some of these assumptions were incorrect, leading to inaccurate assessments of dents. Similarly, Noronha et al. [45] also found that the equations in Appendix R of ASME B31.8 for calculating total strain need revision, and the code should include instructions for measuring dent length. To tackle these concerns, they [46] introduced a new framework for the strain-based evaluation of pipeline dents. This approach employed B-spline curves to interpolate dent geometries and gauge the strain components via in-line inspection (ILI) data. A similar study by Okoloekwe et al. [47] discussed the limitations of the ASME B31.8 strain model in assessing the severity of dents in pipelines. It proposed a novel strain-based approach to assess dents, which involved modifying the strain calculation equations in ASME B31.8 code to capture the three-dimensional (3D) strain state at the dent. In another work by Okoloekwe et al. [48], they proposed a deterministic strain-based approach that utilized multi-dimensional B-spline functions and low-pass filters to smooth the raw ILI signals to evaluate pipeline dents. The proposed methodology provided a rapid and accurate depiction of the extent of dent deformations, while requiring fewer computational resources. Zhang et al. [49] introduced a comprehensive methodology for evaluating geometric strain at the dented area, using ASME B31.8 guidelines and 3D measurements from ILI tools. The method included signal noise filtering, curvature, arch length calculations, and actual strain evaluation.

**Table 4:** Recommended dent strain estimation model in ASME B31.8 code [43].

Description	Expression
Bending strain in the circumferential direction.	$\varepsilon_{1}=\frac{t}{2}(\frac{1}{R_{0}}-\frac{1}{R_{1}})$
Bending strain in the longitudinal direction.	$\varepsilon_{2}=\frac{t}{{2R}_{2}}$
Extensional strain in the longitudinal direction.	$\varepsilon_{3\;}=(\frac{1}{2}){\left(\frac{d}{L}\right)}^{2}$
Strain for the inside and outside pipe surfaces.	$\varepsilon =\frac{2}{\sqrt{3}}\sqrt{\varepsilon_{1}^{2}+\varepsilon_{1}\left(\varepsilon_{2\;}+{\;\varepsilon }_{3}\right)+{\left(\varepsilon_{2}+{\;\varepsilon }_{3}\right)}^{2}}$

where t is the pipe’s wall thickness; $R_{0}$, $R_{1}$, and $R_{2}$ denote the radius of an undamaged pipe and the curvature radius of the indentation within the pipe along the circumferential and longitudinal orientations, respectively; and d and L are dent depth and length, respectively (Figure 11).

More works focused on the local strain response of dented pipelines under indentation loads. Suganuma et al. [50] discuss the effects of local deformation, dent, and strain hardening on the pipe’s compressive strain capacity, finding that a 3 %*D* depth dent reduced the strain capacity by 25%. Huang et al. [51] conducted a numerical simulation investigation on the strain response of constrained dented pipelines subjected to working pressure and assessed the parameter effects. The results highlight the importance of dent depth and proximity to the pipe’s inner wall in determining the severity of the maximum equivalent strain. Their other work [52] developed a multi-physical field coupling model based on FE analysis to explore the mechano-electrochemical (M-E) interaction at a dent-corrosion defect on pipelines. This study provided insights into how the M-E interaction affects the strain response at the defect. Zhu et al. [53] employed the FE method and indentation tests to explore the strain evolution process at a dent–weld defect. The findings of the work suggest that the depth of a dent at the weld, exceeding 3% of the pipe’s outer diameter, should be repaired or replaced. Zhao et al. [54] installed strain gauges along the axial direction of the pipeline at intervals of 50 mm during the full-scale indentation test to obtain the experimental measurement of strain components at the dent region. After that, a twice-elastic slope criterion was utilized to evaluate the plastic collapse strain of dented pipes. Shuai et al. [55] explored the strain historical behavior of a dented pipeline that underwent incremental operating pressure through a burst test and the FE method. The findings indicate that in the case of unconstrained dents, the strain level at the dent varied abruptly and significantly, raising the possibility of fatigue failure under operating pressure. Ghaednia et al. [56,57] explored the impact of concentrated loads on the pipe’s strain distribution, with a focus on the effects of dent length, depth, and operating pressure.

**Figure 11:**
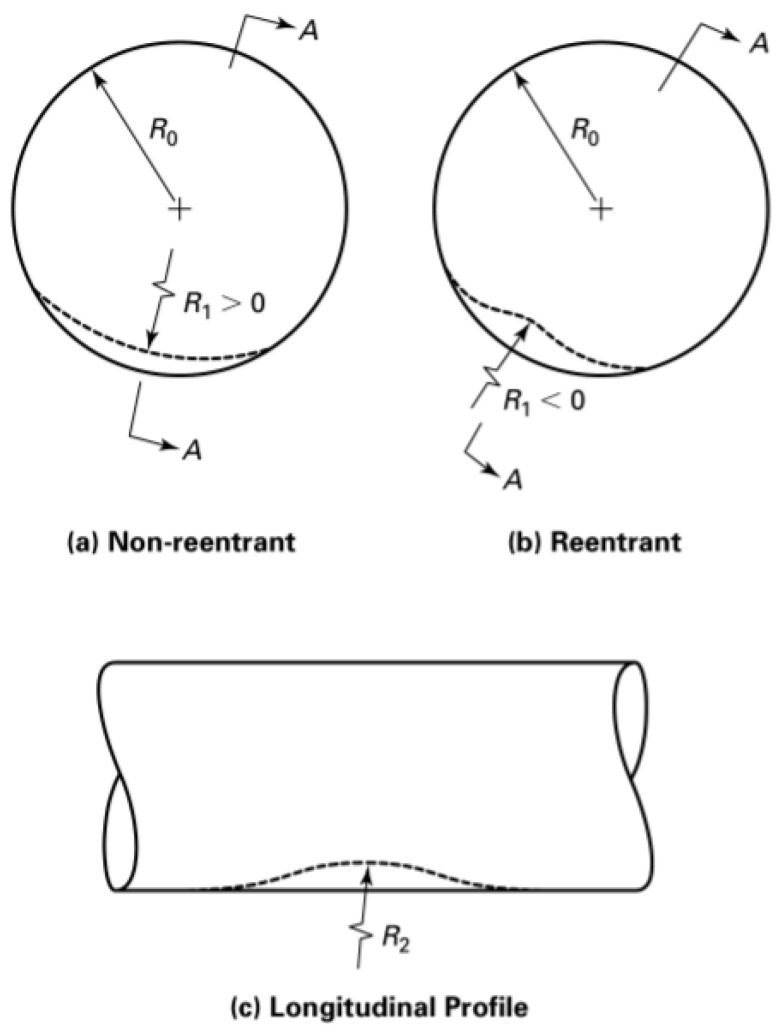
Dent strain estimation model in ASME B31.8 code [43].

#### 3.2.3. Damage-Based Dent Assessment on Steel Pipelines

Damage or ductile damage is the accumulation of plastic strain that a material undergoes [58]. Thus, damage-based dent evaluation criteria can be considered as an expansion of the strain-based criteria [59]. Table 5 shows some damage models for dented pipelines. For example, Alashti et al. [58] assessed the structural-bearing capacity of an aluminum pipe with a dent defect subjected to working pressure using three different ductile fracture models. After comparing the models, it was concluded that the Xue and Wierzbicki (X-W) model is the most reliable in predicting fracture initiation. Building on this finding, Alashti et al. [59] conducted a second study to analyze the damage level of dented aluminum pipes using the X-W model. The outcomes showed that ductile damage significantly affected the pipe’s load-bearing capacity, and the adopted damage model accurately predicted the ductile fracture of dented pipes under multiaxial stress loadings. Their most recent work [60] explored the impact of ductile damage on the mechanical response of dented pipes under operating pressure through experimental and numerical techniques. Specifically, the X-W model was employed to demonstrate its utility in predicting the pipe’s ductile fracture under multiaxial stress conditions. The study’s findings emphasize the importance of considering damage in determining the load-bearing capacity of pipelines with dents. Arumugam et al. [61] adopted a ductile failure damage indicator (DFDI) model to assess the rock-induced pipe bottom dents. The DFDI model was validated through full-scale indentation testing, which allows for the development of strain thresholds for plain dents. Li and Dang [62] examined the ductile damage of pipelines with constrained and unconstrained dents based on the DFDI model and investigated the influence of constraint on pipeline behavior. The results indicate that the crack originated from the interior walls of the pipeline, and the cumulative damage of constrained dents depended on the initial displacement loads and internal pressure. Similarly, Zhao et al. [63] developed a DFDI-based method with an improved strain determination model for assessing pipeline dents. Plata Uribe et al. [64] proposed a stress-modified critical strain (SMCS) criterion for predicting ductile failure of dented pipelines subjected to tensile loading. The proposed approach was validated through full-scale cyclic bending tests, which matched well with experimental data. Wu et al. [65,66,67,68] incorporated the Oyane ductile fracture criterion to evaluate the dented pipelines. They utilized the Oyane criterion and FE calculations to analyze the ductile damage caused by various dents.

#### 3.2.4. Critical Analysis

From the literature on dent evaluation criteria, we found that the most widely used dent evaluation criteria currently include depth-based, strain-based, and damage-based methodologies. However, these metric-based criteria have their merits and limitations: (1) The depth-based dent assessment criterion is simple and easy to apply since the depth of a dent is a straightforward and measurable metric. Currently, 6% of the outer diameter is widely used as the critical dent depth. However, the depth-based criterion is inadequate as it fails to consider the effect of dent geometry [69], pipeline size, and other factors on the damage caused by dents. As a result, it may not accurately reflect the true extent of damage to the pipeline; (2) The Strain-based dent assessment criterion has proven to be superior to the depth-based criterion due to its consideration of the strain distribution in the vicinity of the dent as well as the material properties of the pipeline. Thus, the criterion offers a more precise assessment of pipeline conditions. The prevailing dent strain threshold is 6%. However, the widely adopted equation available for calculating the strain at the dent, recommended by Appendix R in ASME B31.8 code, suffers from a fundamental flaw: it assumes that the pipe wall is in a plane strain state within the dent region. In addition, the definition and determination of dent length are not clear, and the scope of application is limited to smooth dent, without considering fatigue and combined dent. The above limitations may result in an underestimation of dent severity, as the calculated strain may not accurately reflect the true strain state of the dented pipeline. Other, more intricate methods for calculating dent strain require advanced numerical modeling or experimental knowledge, which poses a challenge to the application of strain-based criteria in the dent assessment field; (3) The damage-based criterion provides a more accurate assessment of a dent, as it takes into account the cumulative effect of strain and other factors on the safety of the pipeline. Although the damage-based criterion is the most comprehensive, it demands sophisticated modeling techniques and substantial computing resources. There is currently no universally accepted model for assessing dent damage. The diverse damage assessment models employed by researchers possess their own advantages and disadvantages and may only apply to specific pipeline conditions. The accuracy of damage-based models may require extensive experimental validations.

### 3.3. RQ3: From What Perspective Has the Integrity of Dented Steel Pipelines Been Predicted, and What Research Approaches Have Been Used?

**Figure 12:**
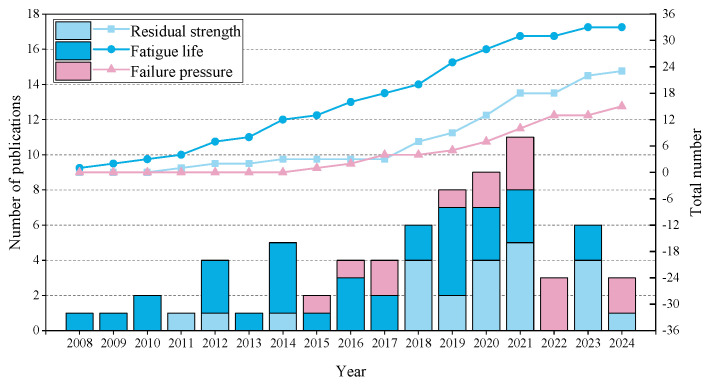
Annual publications.

Due to the potential threat of dents to the safe service of pipelines, accurate integrity evaluation of dented pipelines has become crucial. The review findings of selected 112 publications showed that researchers focused on estimating the remaining strength, fatigue life, and failure pressure of dented pipelines to determine their integrity. The year-wise trend of publications assessing the remaining strength, fatigue life, and failure pressure (burst pressure) of dented pipelines is shown in Figure 12, where the line with markers represents the cumulative number of publications, and the bar chart indicates the annual publication count. As is clear, the annual publication of studies evaluating the fatigue life of dented pipelines reflects the prevalence of fatigue failure in such pipelines. A recent increase in publications estimating these pipelines’ remaining strength and failure pressure emphasizes their importance in integrity research.

#### 3.3.1. Remaining Strength Estimation

Remaining strength denotes the ultimate load that a pipeline can withstand after being subjected to defects or other deterioration effects. When dent defects occur, it becomes critical to evaluate and quantify their effect on the pipe’s load-bearing capacity. As an illustration, Cai et al. [70] developed a quantitative framework for predicting the remaining strength of dented pipelines under bending moments via validated numerical modeling and proposed an empirical formula for the ultimate bending moment. Subsequent studies by the same team [71,72] expanded this framework by using FE models to analyze the ultimate bending-bearing capacity and critical curvature of intact and dented pipelines, particularly emphasizing the severe impact of combined defects (dents associated with gouges). The results show that the coexistence of dent and gouge can lead to a 20–25% reduction in the pipe’s structural strength compared with intact pipelines. In contrast, an isolated gouge only results in a strength decrease of 2–12%, which varies depending on the gouge depth. When a dent is superimposed onto a pre-existing gouge, an additional 8–15% reduction in structural strength is observed.

More studies have highlighted the significance of specific dent parameters, such as length, depth, and geometry, in determining the remaining strength of pipelines. Cai et al. [73] demonstrated that the dent location (compression vs. tensile side) drastically reduced the pipe’s ultimate bending-bearing capacity. Similarly, Wang et al. [74] linked the depth of the kinked dent to the degree of reduction in bending resistance, while Baek et al. [75] emphasized that dent geometry and bending mode are critical factors in the pipe’s ultimate bending-bearing capacity. Shuai et al. [76], Hou et al. [77], and Sun [78] further quantified the effects of dent size, internal/external pressure, and hardening exponents on the pipe’s buckling resistance, and proposed prediction equations. Kainat et al. [79] revealed that dent depth alone is insufficient for evaluating the severity of dents; instead, loading sequence and pressure-cycling history must be considered. This finding emphasized the importance of accurately evaluating the remaining strength of dented pipelines by comprehensively considering operating conditions, defect interactions, and material behavior.

Additional studies have examined the impact of dents on pipeline stability and collapse behavior. Chen et al. [80,81] used nonlinear FE modeling to analyze the collapse and buckling failure of dented pipelines under internal pressure and bending moments. Similarly, Yuan et al. [82] investigated the impact of dents from falling objects on the plastic responses and stability of mechanically lined pipes. Han et al. [83] studied longitudinal, transverse, and tilt dents, revealing that plastic deformation primarily occurs at the dent, influenced by the pipe’s diameter-to-thickness ratio and indenter displacement. These works emphasize the interdependence of multiple variables in the collapse failure mechanism of dented pipes. Zhang and Cheng [84,85] developed a FE model from the perspective of hydrogen-related failure and degradation to demonstrate how dents alter the stress/strain field and hydrogen accumulation in X52 steel pipes. Their findings highlight hydrostatic stress, plastic strain, and initial hydrogen concentration as competing factors influencing hydrogen distribution, which is critical for the remaining strength prediction of pipelines transporting hydrogen. Murugathasan et al. [86] and Wang et al. [87] explored the residual stress and fatigue failure of dented pipelines through FE analysis and burst tests, by linking stress history to load-bearing capacity. Shuai et al. [88] and Wu et al. [89] experimentally investigated the residual stress state during dent formation, while Naghipour et al. [90], Ramasamy and Ya [91], and Limam et al. [92] proposed the framework for evaluating the collapse pressure and bending strain capacity of dented pipelines, integrating theoretical, numerical, and experimental insights.

#### 3.3.2. Fatigue Life Prediction

Fatigue life prediction concerns determining the duration of cyclic loading that a pipeline may endure before yielding to deterioration or damage and falls within the scope of research on remaining life prediction. Unconstrained dents may experience multiple springback or re-rounding due to internal pressure fluctuations, potentially leading to fatigue failure of the pipe wall at the defect. Thus, accurately predicting the fatigue life of dented pipelines is of utmost importance. Many studies have combined experimental methods or numerical simulations to validate the fatigue life prediction for dented pipes. Freire et al. [93] employed non-destructive infrared inspections and strain measurement to forecast the pipe’s fatigue life experimentally. Pournara et al. [94] evaluated the stress concentration factors (SCFs) of dented pipes using cyclic loading tests and parametric numerical studies. Polanco-Loria and Ilstad [95] experimentally examined the fatigue behavior of dented pipelines and introduced a novel SCF equation validated via the fatigue model. Azadeh and Taheri [96,97,98] conducted experimental investigations on the cyclic bending and axial loading effects of dented pipelines and proposed empirical equations for ovalization and ratcheting behavior. Chatziioannou et al. [99] numerically simulated an offshore pipeline subjected to external pressure and cyclic axial loading. Kec and Cerny [100] analyzed the stress–strain behavior and fatigue resistance of dented pipelines through cyclic loading experiments. Tiku et al. [101] developed a numerical model validated by full-scale cyclic fatigue tests and proposed a fatigue life model for dented pipes based on the FE outcomes. Bolton et al. [102] compiled a database of full-scale dent fatigue tests covering field-relevant dent types.

Some studies have explored specific factors that affect the pipe’s fatigue life, such as dent geometry, environmental conditions, or combined defects. Al Nutifat et al. [103] studied the effect of dents and dent–crack combinations on fatigue life. Paiva et al. [104] investigated the effect of environmental conditions on the fatigue life of dented pipes. They measured the cyclic strain amplitude at the dent using the digital image correlation (DIC) technique. Durowoju et al. [105] examined the influence of pipe geometry, material strength, and pressure cycling on SCFs (*K_t_*). Zhu and Wang [106] emphasized the role of residual stress and plastic deformation history in the prediction of dent fatigue life. Hanif and Kenny [107] explored the impact of the coupling of dents and corrosion on the pipe’s fatigue life using strain-based empirical tools. Al-Muslim and Arif [108,109] highlighted the effect of pipe material properties and combined loading on dent fatigue life. Freire et al. [110] combined experimental and numerical data for the fatigue durability evaluation of dented pipelines.

The criticism of existing standards has promoted the improvement of fatigue life prediction models. Alexander and Jorritsma [111] applied the API 579 Level 3 method to estimate SCFs and fatigue life of dented pipes. Shirband et al. [112] criticized the conservatism of the API 579 Level 2 method and proposed improvement suggestions. Zhu [113,114] compared the API 1183’s dent assessment method, revealing inconsistencies and suggesting enhancements. Liu et al. [115] emphasized the consideration of dynamic loading on pipeline dents under highway crossing in the API 1183 code. Pournara and Karamanos [116] developed the guideline for dent repair using numerical results.

Additional studies have employed novel methodologies for predicting dent fatigue life. Garbatov and Soares [117] conducted a fatigue reliability analysis of pressurized pipelines with dents using the Weibull model. Gholami et al. [118] presented a novel approach for fatigue life prediction based on Smith–Watson–Topper criterion validated via FE results. Pinheiro et al. [119,120,121] developed FE models and analytical expressions to predict SCFs, modifying the standard S-N curves for fatigue life assessment. Cunha et al. [122] introduced a stress-life fatigue theory-based algorithm, which was validated through small-scale tests on dented pipes. Turnquist and Smith [123] introduced a life-cycle approach that combines fatigue damage accumulation to estimate the failure probability of dented pipelines. Fontanabona et al. [124] used the Dang Van theory and the Empreinte FE tool to calculate the fatigue life of dented pipelines under cyclic loading. Arumugam et al. [125] developed a fatigue life prediction framework for cracks in dents using stress intensity factors and failure modes.

#### 3.3.3. Failure Pressure Evaluation

Failure pressure evaluation involves predicting the maximum pressure that a pipeline can endure before experiencing rupture failure, that is, studying the ultimate burst pressure of pipelines. Given that internal pressure is the primary load of a pipeline in service, it is imperative to evaluate how the presence of dent defects influences the pipe’s failure pressure. For this purpose, we reviewed the research on failure pressure prediction of dented pipelines specifically in this section. Some studies have employed the FE method, extended finite element methods (XFEM), or analytical equations to predict the failure pressure of dented pipelines. For instance, Gholami et al. [126] and Tian and Zhang [127,128,129] developed FE models and failure criteria based on parameters like maximum equivalent plastic strain (MEPS). Zhao et al. [130] and Tian and Lu [131] combined experimental and numerical methods to analyze the pipe’s burst pressure, while Okodi et al. [132] used XFEM to simulate the crack propagation in dented pipelines. These works focused on numerical or theoretical frameworks to quantify the failure mechanism and derive the prediction equation for the failure pressure of dented pipelines.

Several research studies have investigated the combined effects of multiple defects, such as dent–crack and dent–corrosion, on a pipe’s failure pressure. Huang et al. [133] and Zhao et al. [134] studied how dents and corrosion interact to reduce pressure-bearing capacity, while Ghaednia et al. [135,136] quantified the critical influence of crack depth on limit pressure. Okodi et al. [137] highlighted the impact of increased risk of cracks in dent flanks on the pipe’s burst pressure. Tang et al. [138] used full-scale experiments to verify the effectiveness of the failure assessment diagram method in predicting the burst pressure of pipelines with dent–crack defects. These studies emphasize that combined defects often lead to synergistic degradation of the pipe’s failure pressure. He and Zhou [139] developed a burst pressure prediction model using a deep learning model (TGAN) and a random forest algorithm, demonstrating its superior accuracy over traditional semi-empirical models. This study underscored the potential of data-driven techniques in addressing the limitations of conventional engineering frameworks, especially in the case of limited datasets.

Additional studies have developed tailored predictive formulas for specific dent configurations. Gu et al. [140] categorized dents (constrained vs. unconstrained) and derived formulas for the ultimate internal pressure. Sun et al. [141] determined the effect of critical distances between dents and corrosion defects on the failure pressure. These works prioritized dent-specific characteristics to refine the failure pressure prediction. Allouti et al. [142] found that gouge–dent defects in A37 steel pipelines did not reduce the burst pressure, leading to a new criterion combining volumetric methods and stress triaxiality. Liu et al. [143] validated the stress–strain failure criterion for API 5L X60 pipelines and introduced an efficient failure pressure prediction equation. These findings highlight the need for material- and condition-specific failure pressure prediction methods rather than generalized models.

#### 3.3.4. Critical Analysis

From the literature, we found that over the past two decades, researchers have focused on finding more accurate and reliable methods to predict the mechanical response and failure behavior of pipelines with dent defects under different load conditions, including fatigue, buckling, and collapse. Researchers evaluated the remaining strength, fatigue life, and failure pressure of dented pipelines using theoretical modeling, experimental testing, numerical simulation, or any combination of these approaches. Current research suggests that (1) Dents exhibit a significant impact on the remaining strength (including ultimate bending-bearing capacity, stability, and collapse behavior) of pipelines. The extent of degradation varies based on the type of dent. Researchers have emphasized the significance of dent geometric parameters in determining the pipe’s remaining strength and developed semi-empirical equations to estimate the remaining strength of dented pipelines across diverse loading conditions, but with reduced accuracy and limited applicability. (2) The principal failure mode of pipelines with plain dents is fatigue failure. This is attributed to the cyclic loading resulting from internal pressure fluctuations within the pipeline. The dent-induced local stress concentration escalates the strain range experienced by the pipeline during each pressure cycle. The repetitive strain fluctuations initiate and propagate fatigue cracking. A substantial body of literature has extensively explored methods for forecasting the fatigue life of dented pipelines exposed to cyclic loading. However, the present research is affected by the lack of standardization in dent characterization and a shortage of fatigue-related experimental data. As a result, the proposed prediction models for fatigue life suffer from inadequate precision and applicability. (3) The impact of plain dents on a pipe’s burst pressure is negligible, but the combination of dents with other types of defects can significantly reduce the failure pressure of pipelines. Notably, the combination of dents with metal loss defects like corrosion and gouges may trigger both the curvature change and wall-thinning of the pipeline. This highlights the importance of considering such factors when investigating the dent-related failure pressure. Current studies also face challenges, such as unclear failure mechanisms of combined defects, limited experimental data on high-strength pipelines, and the need for further validation of numerical models.

Based on the above literature research conclusions and existing challenges, relevant industries have developed targeted evaluation standards for dented pipelines, forming the practical application and supplementary improvement of research results. For instance, API RP 1183 [144] establishes three limit state assessments and requires the characterization of dent shape, constraints, and interactions with corrosion, cracks, or welds, as well as multi-level fatigue screening for pressure cycling, which perfectly corresponds to the conclusions in the literature on the importance of dent geometric parameters, fatigue failure risks, and combined defect effects. In contrast, API 579-1 [145] adopts three evaluation levels: Level 1 (simple depth/geometry screening, away from welds and discontinuities), Level 2 (simplified strain/stress analysis), and Level 3 (detailed FE analysis of complex dents), which not only considers the efficient evaluation of conventional dents, but also provides more accurate analysis methods for the complex dents and combined defect evaluation difficulties mentioned in the literature. This standard classifies sharp or kinked dents as high-risk and evaluates their ductile failure, fatigue, and crack initiation, further responding to research findings in the literature on fatigue failure mechanisms and the impact of high-risk dents.

## 4. Conclusive Remarks

This work systematically reviews 112 peer-reviewed papers and conference proceedings on integrity assessment and reliability prediction of dented pipelines over the past two decades. A bibliometric analysis was performed on the selected publications. This work aimed to investigate widely accepted pipeline dent evaluation criteria and the current research status and perspective of integrity assessment of dented pipelines. BA results revealed that the research on pipeline dents has experienced sustained and rapid growth over the past two decades, with the highest output reaching 56 papers between 2019 and 2024. The decline in citations after 2021 is a temporary result of data lag, rather than a decrease in research interest, reflecting the dynamic development of the field. The research in this field focuses on the safety of dented pipelines under internal pressure and has received support from 260 authors from 105 institutions worldwide. Geographically speaking, China (42), Canada (34), Brazil (10), and the United States (9) are at the forefront of global publications, reflecting their strategic roles in the global oil and gas energy system. The findings of SLR show that depth-, strain-, and damage-based evaluation criteria are commonly employed to assess pipeline dents. Depth-based criteria are simple and easy to apply but lack consideration of the effect of other critical parameters on dents; strain-based criteria provide a more accurate evaluation of pipeline conditions, but the dent strain calculation equation proposed by codes (such as ASME B31.8) has an underestimation flaw, challenging the application of strain-based criteria in dent assessments; and damage-based criteria comprehensively consider the cumulative effect of strain and other factors on pipeline safety, but they require complex modeling techniques and a large number of computing resources, hindering its further promotion and utilization. The reliability of dented pipelines is mainly predicted through theoretical modeling, experimental testing, numerical simulation, or any combination of these approaches from three aspects: remaining strength, fatigue life, and failure pressure. It was noted that dents associated with any other defects could significantly reduce the failure pressure of pipelines, highlighting the importance of considering such factors in pipeline dent studies.

The limitation of this review is that it only used the WoS database, without including other databases such as Scopus, Engineering Village, and Google Scholar; it only includes English peer-reviewed papers and excluded non-English literature, industry reports, patent and engineering data; it did not consider the literature evaluating dents on pipelines containing other media such as hydrogen, methanol, and supercritical carbon dioxide, nor did it involve the literature assessing dents on pipelines transporting these special media; it lacks a clearer form of quantitative or structured comparison of the reviewed models and approaches. In addition, BA lacked citation network analysis and more advanced bibliometric indicators.

Hence, potential future directions for extending pipeline dent studies involve:

(1) The development of higher-fidelity and more robust numerical models capable of characterizing the mechanical response and service behavior of dented pipelines under diverse complex loading scenarios. This includes the representation of realistic loading histories, material constitutive models accounting for environmental degradation induced by transmission media, and precise simulation of complex interfacial interactions and boundary constraints.
(2) Additional full-scale experimental investigations under varied loading conditions, including combined mechanical loads and fluctuating internal pressure, are required to generate comprehensive experimental datasets for the validation and refinement of numerical and analytical models.
(3) The integration of advanced sensing technologies and in-line inspection (ILI) techniques, such as guided ultrasonic waves and intelligent pipeline robots, to enable real-time detection, identification, and localization of pipeline dent defects.
(4) The establishment of industry-accepted ductile damage evaluation frameworks that enable accurate severity assessment of pipeline dents. This necessitates an improved understanding of variability in dent geometry, location, and dimension, as well as the nonlinear mechanical behavior of pipeline materials, geometric characteristics, and formation conditions associated with dent defects.
(5) In recent years, increasing attention has been directed towards pipeline dents occurring in conjunction with other defect types and the synergistic effects among multiple similar or dissimilar dent defects. Further investigation is thus recommended to quantify the influence of interactions between dents and other metal-loss defects, including gouges, corrosion, and cracks, on pipeline structural safety, as such combined defect conditions induce local curvature variations and wall thickness anomalies.

## Figures and Tables

**Table 1:** Keywords adopted in the search strategy.

Primary Terms	Dent	Pipeline	Safety
Derived terms	Dent depth	Oil pipeline	Integrity
Derived terms	Dent strain	Natural gas pipeline	Reliability
Derived terms	Dent damage	Hydrogen pipeline	Ultimate strength
Derived terms	NA	NA	Fatigue life
Derived terms	NA	NA	Failure pressure

**Table 2:** Search strings for retrieving publications related to pipeline dents.

Keywords	Search Strings
Pipeline dents	(((“dent” OR “dented”) AND (“pipe *”)) AND (“integrity” OR “reliability” OR “ultimate strength” OR “fatigue life” OR “failure pressure” OR “limit pressure” OR “depth” OR “strain” OR “ductile damage” OR “plastic damage”))

* serves as a truncation symbol that matches any number of characters and shows all words starting with that root.

**Table 3:** Preliminary screening criteria for literature.

**Inclusion criteria**	Articles that are in the field of engineering
	Articles that are related to pipeline dent assessment
	Articles that predict the reliability of dented pipelines
**Exclusion criteria**	Non-English articles
	Articles that are unrelated to pipeline dent assessment
	Articles that cannot predict the reliability of dented pipelines
	Non-peer-reviewed articles

**Table 5:** A synopsis of the damage models employed in pertinent studies [58,59,60,61,62,63,64,65,66,67,68].

Models	Expression of Damage Models	Ref.
Wilkins’ model	$D=\int_{0}^{\varepsilon_{c}}\frac{{(3-\max\left\{-\frac{S_{3}}{S_{1}},-\frac{S_{1}}{S_{3}}\right\})}^{\lambda }}{D_{cr}{(1+\alpha p)}^{\beta }}d\varepsilon_{eq}^{p}\;\leq \;1$	[58]
Improved Johnson–Cook model	$D=\int_{0}^{\varepsilon_{c}}\frac{1}{\varepsilon_{f}(\frac{\sigma_{m}}{\sigma_{eq}},\varepsilon_{p}^{\cdot },T)}d\varepsilon_{eq}^{p}\;\leq \;1$	[58]
X-W model	$D=\int_{0}^{\varepsilon_{c}}\frac{m}{\varepsilon_{f}}{\left(\frac{\varepsilon_{eq}^{p}}{\varepsilon_{f}}\right)}^{m-1}d\varepsilon_{eq}^{p}\;\leq \;1$	[58,59,60]
DFDI model	$D=\int_{0}^{\varepsilon_{c}}\frac{1}{\beta \cdot \varepsilon_{f}\cdot \exp(-\frac{3}{2}\frac{\sigma_{m}}{\sigma_{eq}})}d\varepsilon_{eq}^{p}\;\leq \;1$	[61,62,63]
SMCS model	$D=\int_{0}^{\varepsilon_{c}}\frac{1}{\alpha \cdot \exp(-\beta \cdot \frac{\sigma_{m}}{\sigma_{eq}})}d\varepsilon_{eq}^{p}\;\leq \;1$	[64]
Oyane’s model	$D=\frac{1}{\beta }\int_{0}^{\varepsilon_{c}}(\alpha -\frac{\sigma_{m}}{\sigma_{eq}})d\varepsilon_{eq}^{p}\;\leq \;1$	[65,66,67,68]

where D, $\varepsilon_{c}$ and $\varepsilon_{eq}^{p}$ denote the damage parameter, critical strain to failure and equivalent plastic strain, respectively; $S_{1}$ and $S_{3}$ are maximum and minimum components of principal deviatoric stress, respectively; $D_{cr}$ and λ are the material constant and function exponent in Wilkins’ model, respectively; α and β are material constants; $\varepsilon_{f}$ is equivalent fracture strain; p is hydrostatic pressure; $\sigma_{eq}$ and $\sigma_{m}$ are mean stress and von Mises stress, respectively; $\varepsilon_{p}^{\cdot }$ is plastic strain rate; T is time; and m is the damage exponent in the X-W model.

## Data Availability

No new data were created or analyzed in this study. Data sharing is not applicable to this article.
